# Supplementary figures and images for: PQN-59 antagonizes microRNA-mediated repression during post-embryonic temporal patterning and modulates translation and stress granule formation in C. elegans
Source: PLoS Genet. 2021 Nov 22;17(11):e1009599. doi: 10.1371/journal.pgen.1009599 (PMC8648105; doi:10.1371/journal.pgen.1009599)

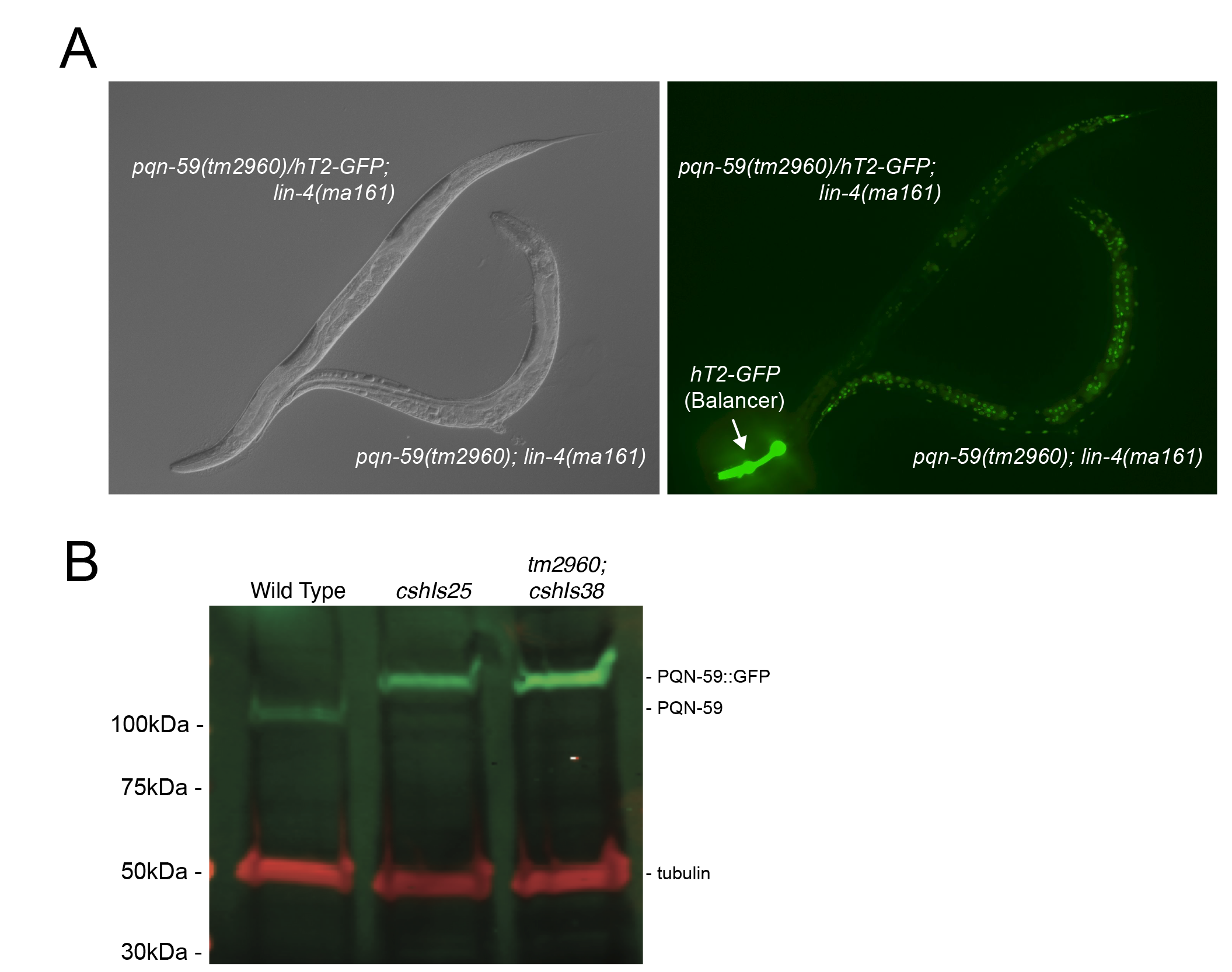

Supplement: S1 Fig — (A) pqn-59(tm2960); lin-4(ma161) animals exhibit wild type col-19::GFP expression (and protruding vulva phenotype) while animals harboring a single copy of the pqn-59 deletion allele (balanced with an hT2 myo-2::GFP balancer) exhibit only a very mild col-19::GFP expression phenotype and no vulval induction. (B) Western blots of wild-type, csh150[PQN-59::GFP CRISPR allele at pqn-59 locus], and pqn-59(tm2960); csh151 [PQN-59::GFP single copy on Chromosome II] animals using antibodies against the PQN-59 amino terminus. (TIF) [file pgen.1009599.s001.tif]

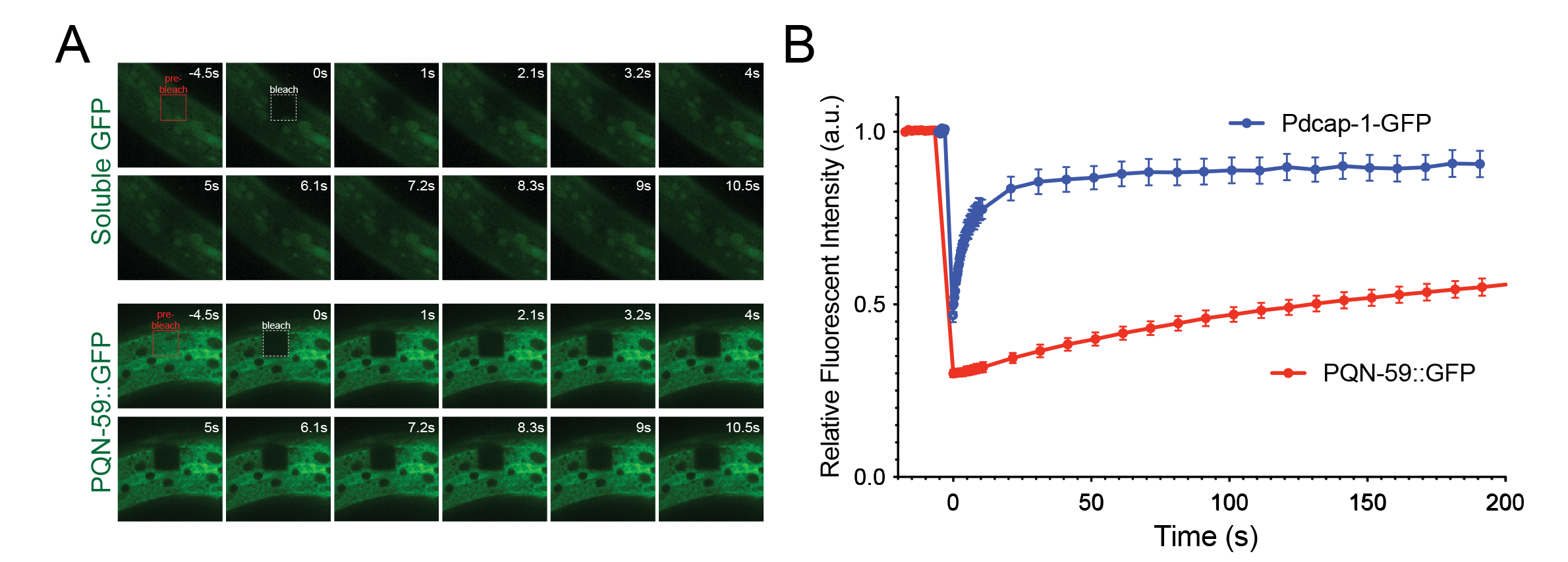

Supplement: S2 Fig — (A) FRAP analysis of soluble GFP (driven from the dcap-1 promoter) and a translational fusion of PQN-59. (B) Quantification of the recovery rates for each GFP protein depicted in A. Graphs represent the average recovery rate and error bars indicate that standard error of measurements (SEM) for 10–15 photobleaching events in separate animals. (TIF) [file pgen.1009599.s002.tif]

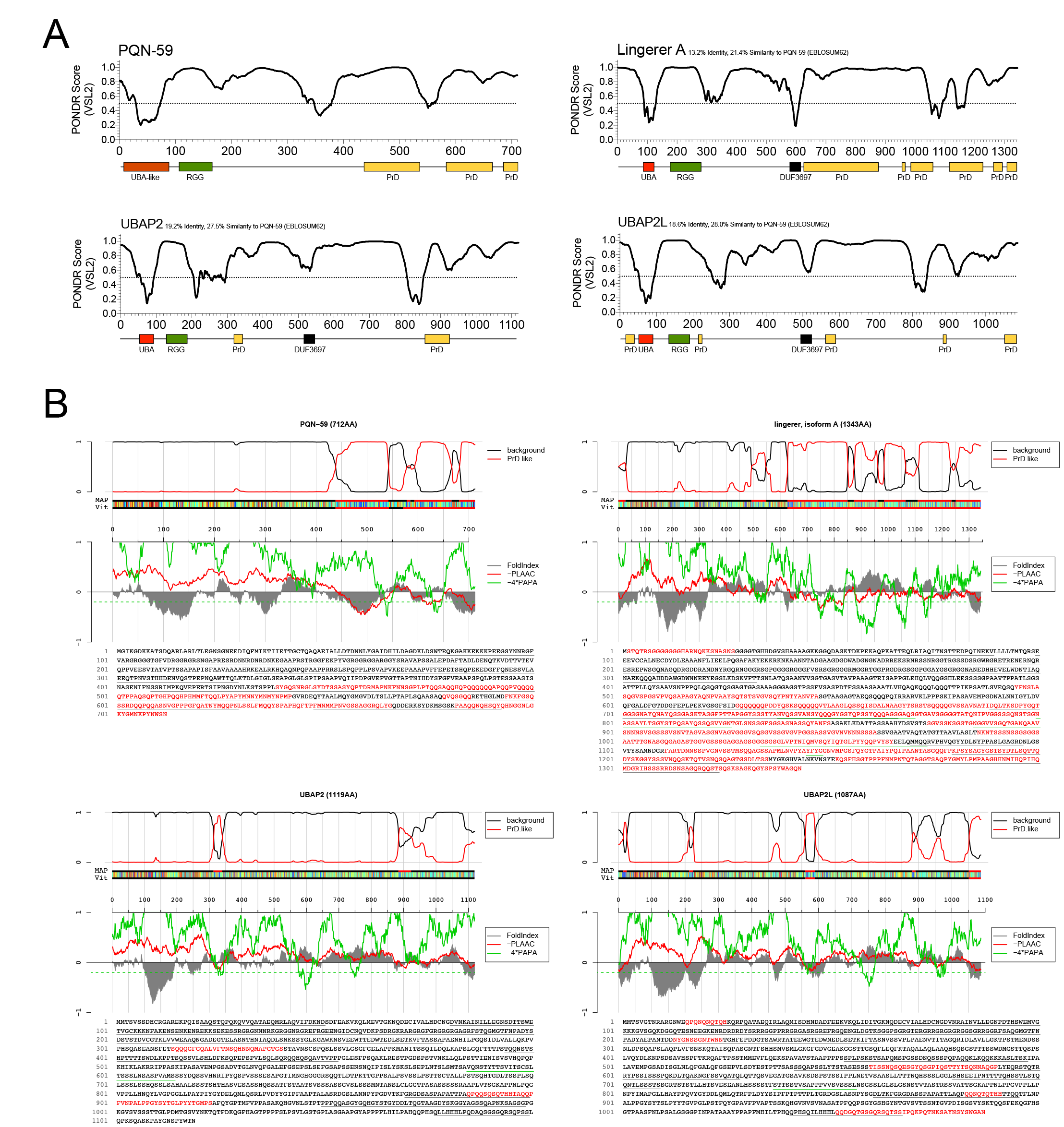

Supplement: S3 Fig — The PLAAC: Prion-Like Amino Acid Comparison Server (http://plaac.wi.mit.edu) was used to query the primary amino acid sequences of PQN-59, Lingerer isoform A, UBAP2 and UBAP2L with the following parameters: Core length of 60 and a relative weighting of background probability set to the corresponding species of origin. In protein sequences below each PLAAC graph, red highlighted amino acids indicate glutamine (Q) and asparagine (N) rich sequences identified by this analysis as encoding “prion-like” domains. (TIF) [file pgen.1009599.s003.tif]

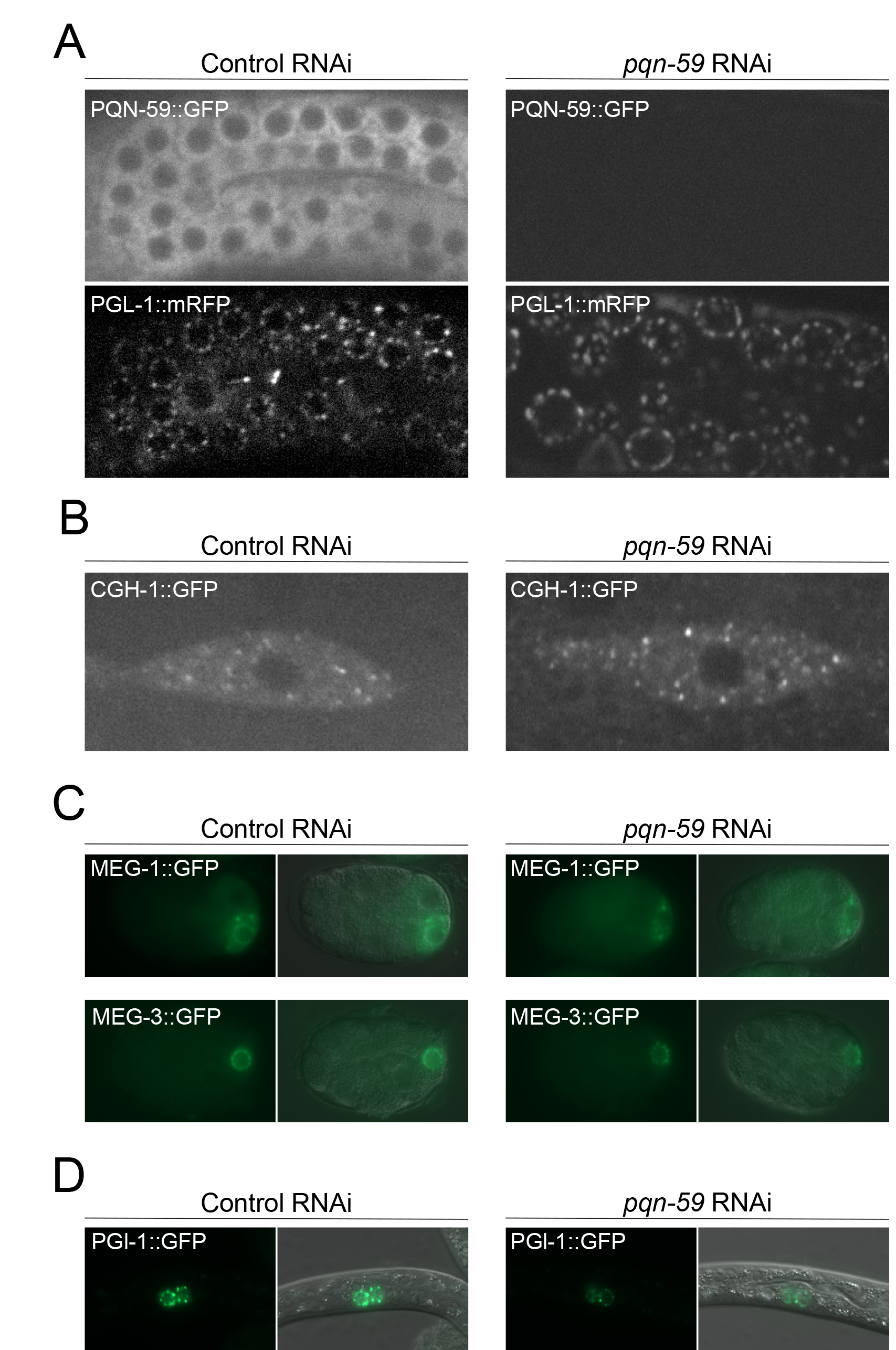

Supplement: S4 Fig — (A) Confocal images of PGL-1mRFP and PQN-59::GFP in late L4 staged F1 animals exposed to control or pqn-59 dsRNAs. (B) Confocal images of CGH-1::GFP expression in the lateral seam cells of L4-staged F1animals exposed to control or pqn-59 dsRNAs. (C) MEG-1::GFP and MEG-3::GFP expression in P cells of F1embryos exposed to control or pqn-59 dsRNAs. (D) PGL-1::GFP expression in early L1-staged F1 animals exposed to control or pqn-59 dsRNAs. (TIF) [file pgen.1009599.s004.tif]

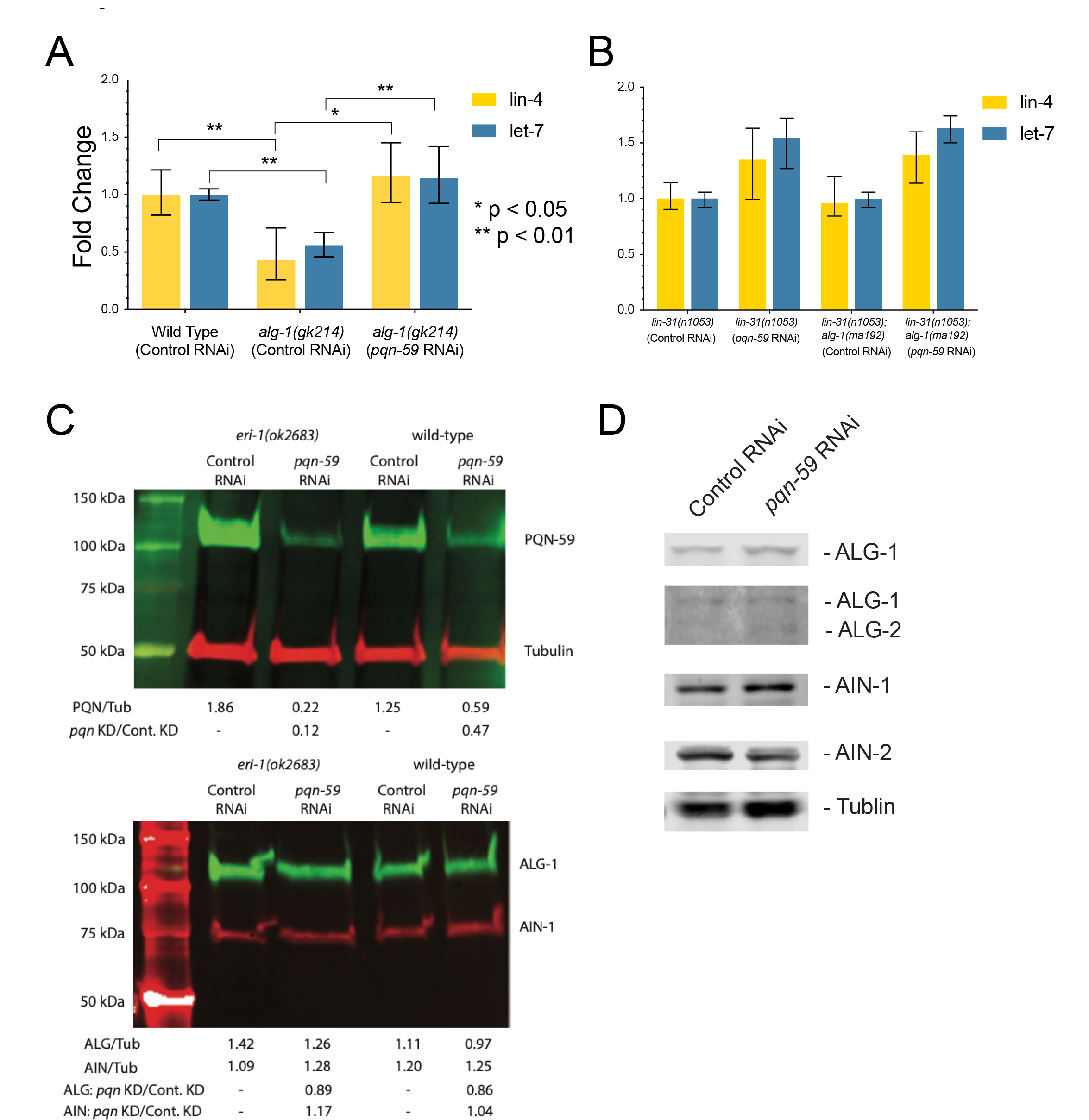

Supplement: S6 Fig — (A) Taqman analysis of lin-4 and let-7 miRNAs isolated from wild-type or alg-1(gk214) animals subjected to control or pqn-59 dsRNAs. In each measurement was standardized by also quantifying the expression of U18 snoRNA in each sample. Error bars represent standard deviation (n = 3 biological replicates, two technical replicates). P-values were calculated using a Student’s t-test, and corrected for multiple comparisons using a Bonferroni correction. (B) Taqman assays as outlined in panel A performed on young adult-staged lin-31(n1053) and lin-31(n1053); alg-1(ma192) animals subjected to control or pqn-59 dsRNAs. (C) In the eri-1(ok2683) RNAi hypersensitive strain pqn-59 depletion decreases PQN-59 to 12% of its abundance in control eri-1(ok2683) animals. In wild-type animals pqn-59 depletion via RNAi decreases PQN-59 to less than half of its abundance in animals fed control RNAi. In contrast, ALG-1 and AIN-1 abundance are minimally affected by pqn-59 depletion. (D) Western blots of miRISC components of N2 animals grown on control or pqn-59 dsRNAs from hatching to adulthood. Western blots were prepared with LiCOR reagents and imaged with a Classic Infrared Odyssey imager. Quantification was performed in ImageJ. (TIF) [file pgen.1009599.s006.tif]
